# Laser speckle flowgraphy in juxtapapillary retinal capillary hemangioblastoma: a case report on natural course and therapeutic effect

**DOI:** 10.18632/oncotarget.27771

**Published:** 2020-10-20

**Authors:** Mizuho Mitamura, Satoru Kase, Kiriko Hirooka, Susumu Ishida

**Affiliations:** ^1^Department of Ophthalmology, Faculty of Medicine and Graduate School of Medicine, Hokkaido University, Sapporo, Japan; ^2^Department of Ophthalmology, Teine Keijinkai Hospital, Sapporo, Japan

**Keywords:** juxtapapillary retinal capillary hemangioblastoma, laser photocoagulation, laser speckle flowgraphy, fluorescein angiography, von Hippel-Lindau disease

## Abstract

Juxtapapillary retinal capillary hemangioblastoma (JRCH), a benign intraocular vascular tumor, is usually progressive and may lead to severe vision loss due to various complications. We herein present a case of JRCH observed with laser speckle flowgraphy (LSFG) before and after laser photocoagulation (LPC). A 21-year-old Japanese woman underwent LSFG evaluations. Right eye showed an orange-colored tumor consistent with JRCH on the papillomacular bundle, where LSFG showed a mild warm-color blood flow signal. Eight months after the first examination, JRCH in the right eye increased redness with vasodilatation, and the size enlarged, where LSFG showed a stronger warm-color blood flow signal. She underwent direct yellow laser ablation for the JRCH lesion. One week after LPC, JRCH became paler and LSFG eventually depicted a weakened blood flow signal at the same site. In conclusion, non-invasive and reproducible LSFG is a useful tool for assessing not only JRCH activity but also therapeutic effect.

## INTRODUCTION

Juxtapapillary retinal capillary hemangioblastoma (JRCH), a benign intraocular vascular tumor, arises on or adjacent to the optic nerve head [1]. JRCH may occur sporadically (54%) or as a manifestation of von Hippel-Lindau (VHL) disease (46%), an autosomal dominant neoplastic disorder [1]. On long-term follow-up of patients with JRCH, the visual acuity generally worsens [2], and may cause various complications such as macular edema, retinal exudation, epiretinal membrane formation, exudative retinal detachment, and subsequent tractional retinal detachment [3]. It is likely that indication of treatments depends on progressive clinical manifestations of JRCH such as enlargement of a tumor size, alteration of tumor coloration, and retinal hemorrhage [3, 4]. Several treatments have been proposed, including laser photocoagulation (LPC), photodynamic therapy (PDT), intravitreal anti-vascular endothelial growth factor (anti-VEGF) therapy, radiotherapy, cryopexy and surgical excision [4]. We have reported that LPC might be the first-line treatment for JRCH [3, 4]; however, it is challenging to objectively evaluate the effects of LPC on changes in blood flow within the tumor tissues and tumor activity.

Laser speckle flowgraphy (LSFG) is a blood flow imaging device based on laser scattering, which non-invasively allows for two-dimensional visualization of fundus circulation in various intraocular mass lesions such as optic disc melanocytoma [5], choroidal macrovessel [6] and sclerochoroidal calcification [7]. However, there are no reports demonstrating LSFG findings in JRCH. We herein report a case of JRCH treated with LPC and show LSFG findings before and after LPC.

## CASE REPORT

A 21-year-old Japanese woman complained of blurred vision in her left eye and was referred to our clinic because of fundus lesions. She was diagnosed with JRCH oculus sinister (OS) at the age of 8 when she was pointed out to have poor visual acuity at school health check. At that time, she underwent brain magnetic resonance imaging and echocardiography, proving no abnormalities. At the doctor’s discretion, her left eye was observed without treatment until age 14, and she suspended her visits. She had a history of schizophrenia with no family history of any other disorders including VHL disease. Her best-corrected visual acuity (BCVA) was 1.0 oculus dexter (OD) and 0.02 with disuse exotropia OS, and her intraocular pressure was normal oculi uterque (OU). Slit-lamp microscopy did not detect any findings OU. The patient received multimodal imaging evaluations including funduscopy, swept-source optical coherence tomography (SS-OCT), fluorescein angiography (FA), and LSFG, together with systemic examinations such as whole body magnetic resonance imaging. The institutional review board of Hokkaido University Hospital waived ethical assessment of this clinical study because of a single case report. This study adhered to the tenets of Declaration of Helsinki.

**Figure 1:**
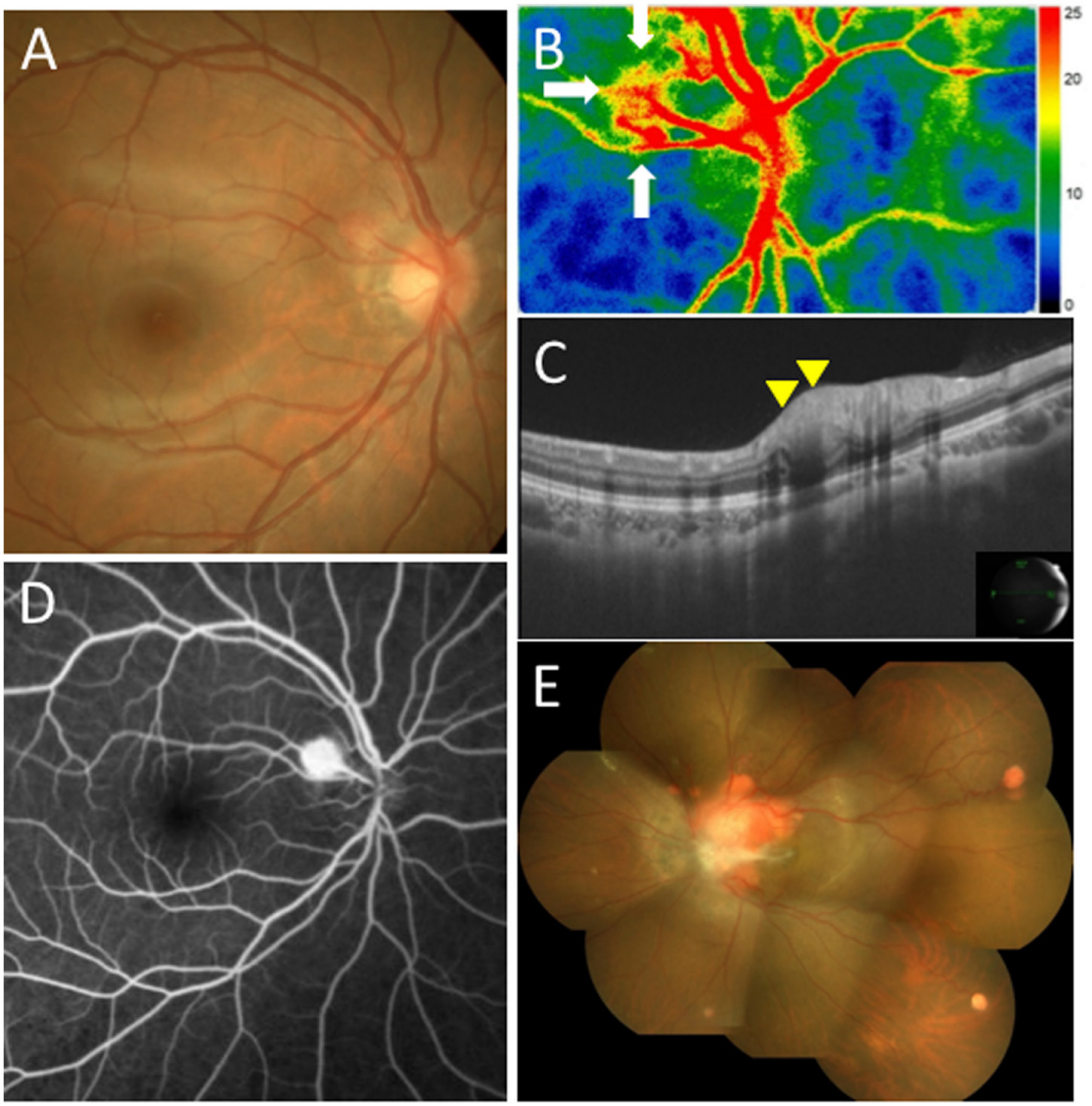
Initial findings on color fundus photography (CFP), swept-source optical coherence tomography (SS-OCT), laser speckle flowgraphy (LSFG), and fluorescein angiography (FA) in the present case with juxtapapillary retinal capillary hemangioblastoma (JRCH). (**A**) CFP in the right eye showing an orange-colored endophytic tumor adjacent to the temporal optic disc. (**B**) LSFG in the right eye showed a mild blood flow signal from the optic nerve to the superotemporal retinal capillary hemangioblastoma (white arrows). (**C**) SS-OCT in the right eye at horizontal scans through the JRCH lesion showing an elevated mass in the superotemporal site of the optic disc (yellow arrowheads). (**D**) FA in the right eye detected hyperfluorescence in the JRCH lesion adjacent to the temporal optic disc in the early phase. (**E**) CFP in the left eye showing multiple yellowish to orange-colored hemangioblastomas mainly near the optic disc with serous retinal detachment in addition to several peripheral tumors.

Color fundus photography (CFP) revealed an orange-colored endophytic tumor adjacent to the temporal optic disc OD (Figure 1A). The tumor size (long and short axes) on CFP was 0.99 × 0.90 mm at her first visit (Figure 1A). LSFG showed a mild warm-color blood flow signal corresponding to JRCH OD (Figure 1B, white arrows). Mean blur rate (MBR), a quantitative index of blood flow velocity on LSFG, was 20.1 arbitrary units (AU). SS-OCT demonstrated a mildly elevated lesion located throughout the inner retinal layers in the horizontal section of the tumor OD (Figure 1C, yellow arrowheads). FA detected hyperfluorescence in JRCH in the early phase (Figure 1D), where dye leakage was noted in the late phase. On the other hand, CFP in the left eye showed multiple yellowish to orange-colored vascular tumors mainly near the optic disc with serous retinal detachment in addition to several peripheral tumors (Figure 1E).

**Figure 2:**
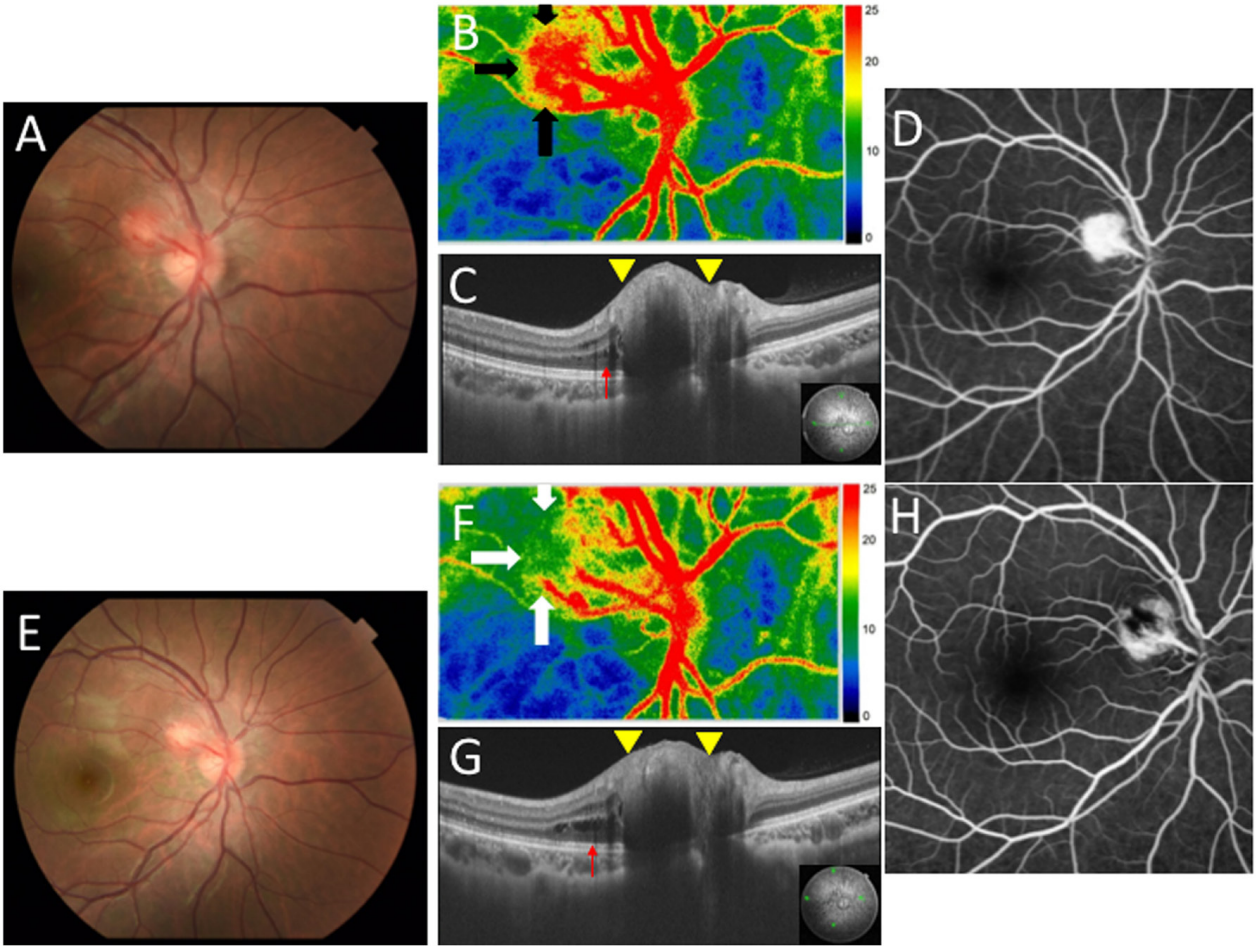
Ophthalmic findings before and after laser photocoagulation (LPC) in the present case with JRCH in the right eye. (**A**) CFP showing the enlarged and dilated hemangioblastoma compared to the initial visit. (**B**) LSFG showed a strong blood flow signal on the superotemporal site of the optic disc (black arrows). (**C**) SS-OCT at horizontal scans through the JRCH lesion showing a more elevated mass in the superotemporal site of the optic disc than that at the initial visit (yellow arrowheads), together with the development of retinoschisis (red arrow). (**D**) FA detected hyperfluorescence in the JRCH lesion in the early phase. (**E**) CFP revealed the paler and smaller hemangioblastoma compared to Figure 2A. (**F**) LSFG showed a weakened blood flow signal on the superotemporal site of the optic disc (white arrows). (**G**) SS-OCT at horizontal scans through the JRCH lesion showing a less elevated mass in the superotemporal site of the optic disc than Figure 2C (yellow arrowheads), together with persistent retinoschisis (red arrow). (**H**) FA detected hyperfluorescence spot around hypofluorescence in the JRCH lesion in the early phase.

A systemic examination revealed multiple vascular tumors involving the spinal cord, medulla oblongata, and kidney. Histopathology of the tumor in the spinal cord revealed hemangioblastoma. Based on the above findings, she was clinically diagnosed with suspected VHL disease. Eight months after the first examination, JRCH became enlarged to 1.64 × 1.36 mm with marked redness due to vasodilatation (Figure 2A). LSFG showed an augmented warm-color blood flow signal on the tumor of the optic disc OD (Figure 2B, black arrows). MBR increased to 24.7 AU. SS-OCT demonstrated a more elevated mass in the superotemporal site of the optic disc OD than at the initial presentation (Figure 2C, yellow arrowheads), together with the development of retinoschisis (Figure 2C, red arrow). FA detected hyperfluorescence in JRCH in the early phase (Figure 2D) with marked dye leakage in the late phase. Her BCVA was unchanged at 1.0.

After informed consent was obtained, she underwent direct yellow laser ablation of the JRCH lesion (25 shots, 0.1–0.3 sec in duration, 50 μm spot size, and 100–150 mW power). One week after LPC, JRCH became paler with its size measuring 1.80 × 1.71 mm (Figure 2E), and a weakened blood flow signal was observed at the same site on LSFG (Figure 2F, white arrows). MBR decreased to 14.0 AU after the treatment. SS-OCT demonstrated a less elevation in the tumor of the optic disc OD (Figure 2G, yellow arrowheads). FA detected a hyperfluorescent spot admixed with hypofluorescence at the JRCH lesion in the early phase (Figure 2H) with reduced dye leakage in the late phase. Serous retinal detachment (SRD) occurred following LPC, causing temporary visual impairment. Her vision gradually recovered with short-term oral administration of corticosteroids, suggesting LPC-associated inflammation as a possible mechanism underlying SRD. Two years after the first diagnosis, her BCVA was 1.2 OD and there was no recurrence of the hemorrhage or enlargement of JRCH.

## DISCUSSION

The present study demonstrated novel findings on JRCH observed with LSFG, so as to better understand blood flow changes in JRCH before and after LPC. To the best of our knowledge, this report is the first to show vascular obstruction and blood flow reduction in JRCH following LPC by means of LSFG monitoring.

In general, the clinical course of JRCH can deteriorate, thereby causing various complications such as macular edema, retinal exudation, epiretinal membrane formation, and retinal detachment [3]. As concerns this case, the complication-related vision loss could have been irreversible because her left eye had already had poor vision. Indeed, there is no definite treatment protocol for JRCH; nevertheless, assessment of JRCH activity is very important in order not to miss the timing of treatment; but nowadays there has been few methods to evaluate the activity. Indeed, no clear consensus has been achieved on the appropriate timing of intervention, although there are several treatment options for JRCH [4]. In this case, LPC was selected because JRCH less than 3 mm was located near the optic disc without SRD. The timing of treatment was also judged from the tendency of tumor size expansion, alteration of color tone, and LSFG findings.

In our case, the right eye was observed without any previous treatments at the first visit when CFP showed an orange-colored tumor and SS-OCT demonstrated a mildly elevated lesion. In addition, LSFG showed a mild warm-color blood flow signal corresponding to JRCH. JRCH was followed up by assessing biomicroscopic, SS-OCT and LSFG findings. After 8 months of follow-up, the color of the tumor increased redness with vasodilatation, the size enlarged on CFP, and the height was elevated on SS-OCT. Although hyperfluorescent findings depicted with FA were not obviously different between before and after the exacerbation, LSFG clearly detected an enhanced warm-color blood flow signal at this time. This means that LSFG more clearly showed hyperperfusion signals when the tumor activity worsened, in contrast to FA findings. Taken together, the blood flow signals on LSFG changed with time during the exacerbation, indicating that LSFG was important for evaluating tumor activity.

In our case, FA detected hypofluorescent spots in the LPC-treated area, which coincides with the low blood flow area on LSFG. Although FA findings were consistent with LSFG findings, it was unclear to what extent the tumor body had lost blood flow, due to hyperfluorescence surrounding hypofluorescence. Indeed, Sagar et al. described the shortcomings of FA as follows: tumors captured in the early phase of FA show well-defined borders, whereas tumors captured in the late phase are ill-defined due to obscuration of margins by dye leakage [8]. In contrast, LSFG findings before and after LPC in our case could clearly show a decrease in blood flow, indicating that LPC induced hypoperfusion within the tumor tissue. LSFG has been shown favorable reproducibility for the evaluation of ocular circulation, particularly in the optic disc and choroid [9]. Taken together, the non-invasiveness and reproducibility of LSFG are valuable not only for assessing tumor activity but also for therapeutic effect.

In addition to FA, OCT angiography (OCTA) has been increasingly regarded as a useful tool to monitor response to JRCH treatment [8, 10–12] and identify harmless nonvascular lesions in VHL disease [8]. JRCH tissues were evaluated with OCTA that showed a reduced blood flow directly after LPC, which visualized the intratumor perfusion as partially blocked [10]. However, the shortcomings of OCTA included the presence of motion artifacts and segmentation errors, thus leading to degradation of image quality and duplication of lesions [10]. Compared with the evaluation with FA and OCTA, this case report showed clear dynamic alterations of blood flow on LSFG before and after treatment, although it would be difficult to depict morphological alterations with LSFG.

Besides LPC, there are several other treatment options for JRCH such as intravitreal anti-VEGF therapy and PDT [4]. As concerns anti-VEGF therapy, the overall results indicated limitations of intravitreal anti-VEGF treatment as monotherapy: first, the effective dose required for JRCH may be higher than that for neovascular age-related macular degeneration; second, the route of administration through the vitreous may limit the access to tumor cells in the endophytic lesions [13]. PDT was shown to be effective in causing fibrosis and involution of relatively small JRCH lesions, while it remains unknown whether PDT contributes to the treatment of large tumors. Moreover, there are no previous reports on the mechanisms underlying therapeutic efficacy of anti-VEGF therapy or PDT for JRCH tissues. In this study, LSFG revealed that LPC could reduce blood stream, thereby disrupting the tumor activity in JRCH. It is a future task to prove how LSFG would demonstrate the effects of other treatments so as to determine the optimal intervention in the long-term management of JRCH.

As for histopathological findings, JRCH is known to be composed of endothelial cells, pericytes, and foamy stromal cells [4]. The protein VHL (pVHL), the VHL suppressor gene product, is known to downregulate VEGF expression. With the absence of pVHL, VEGF is upregulated, resulting in neovascularization on and around JRCH. Chan et al. showed that the stromal cells of the retinal hemangioblastoma have a complete loss of VHL gene and enhanced VEGF gene expression [14]. Clinically, the goal of treatments for JRCH should be induction of tumor regression and/or selective vascular occlusions by minimally invasive procedures, because JRCH is located near the papillomacular bundle. Although there is no previous report on how LPC is effective for JRCH, it is considered that the LPC treatment physically destroyed the stromal cells within the tumor tissues, reduced VEGF expression, and caused vascular occlusion leading to tumor regression. These mechanisms were consistent with the currently observed decrease in blood flow of tumor tissues based on LSFG signal alterations.

In conclusion, we reported a case of JRCH treated with LPC and observed LSFG findings at baseline and before and after LPC. LSFG is useful for evaluating not only tumor activity but also therapeutic efficacy, which may play an important role in the management of JRCH.
