# Addendum: Laser speckle flowgraphy in juxtapapillary retinal capillary hemangioblastoma: a case report on natural course and therapeutic effect

**DOI:** 10.18632/oncotarget.28297

**Published:** 2023-05-26

**Authors:** Mizuho Mitamura, Satoru Kase, Kiriko Hirooka, Susumu Ishida

**Affiliations:** ^1^Department of Ophthalmology, Faculty of Medicine and Graduate School of Medicine, Hokkaido University, Sapporo, Japan; ^2^Department of Ophthalmology, Teine Keijinkai Hospital, Sapporo, Japan

**This article has an addendum:** Informed patient consent was obtained.

Original article: Oncotarget. 2020; 11:3800–3804. 3800-3804. https://doi.org/10.18632/oncotarget.27771

